# Acute Caffeine Administered in Chewing Gum May Improve Functional Firefighting Balance After Gamma-Aminobutyric Acid Supplementation: A Double-Blind Crossover Trial

**DOI:** 10.3390/nu18172876

**Published:** 2026-09-02

**Authors:** Mei-Hui Tseng, Ching-Lin Wu, Yi-Jie Shiu, Yi-Chun Tsou, Hsien-Tsung Lin, Chih-Hui Chiu

**Affiliations:** 1 Institute of Biomedical Sciences, National Chung Hsing University, Taichung 402202, Taiwan; tsengmh2009@gmail.com; 2 Graduate Institute of Sports and Health Management, National Chung Hsing University, Taichung 402202, Taiwan; psclw@dragon.nchu.edu.tw; 3 Physical Education and Sport Sciences, National Taiwan Normal University, Taipei 106308, Taiwan; shiu880511@gmail.com; 4 Graduate Program in Department of Exercise and Health Science, National Taiwan University of Sport, Taichung 404401, Taiwan; tsou.9008@gmail.com (Y.-C.T.); sp20196@gmail.com (H.-T.L.)

**Keywords:** caffeine, heart-rate variability, α-amylase, sympathetic nervous system, firefighting balance

## Abstract

Background: This study investigated whether caffeinated chewing gum enhances sympathetic activity and improves functional firefighting balance performance after gamma-aminobutyric acid (GABA) supplementation. Methods: Fourteen active-duty elite firefighters (age: 36 ± 5 years; height: 174 ± 5 cm; BMI: 25.2 ± 1.6 kg/m^2^) participated in a double-blind randomized crossover trial. The participants completed functional firefighting balance tests while wearing approximately 25 kg of full firefighting gear. After a 1 h resting period, the participants ingested GABA and rested for an additional 15 min before chewing either caffeine-containing gum (CAF) or caffeine-free gum (CG) for 10 min. Fifteen minutes after gum ingestion, the participants repeated the functional firefighting balance tests. Completion time and performance index were used as the indicators of the functional firefighting balance tests. Results: The CAF trial demonstrated a significantly better completion time (*p* = 0.011; Cohen’s d = 0.54) and performance index (*p* = 0.012; Cohen’s d = 0.43) compared with the CG trial, with small-to-moderate effect sizes. There were no significant interactions in HR, SDNN, RMSSD, LF and HF (condition*time, *p* > 0.05). There was a significant interaction in LF/HF (*p* = 0.040); however, further statistical analysis revealed no significant differences across trials or over time (*p* > 0.05). Salivary α-amylase concentration was also significantly higher in the CAF trial compared with the CG trial after testing (*p* = 0.040; Cohen’s d = 0.45). Conclusions: These findings suggest that caffeinated chewing gum may enhance functional balance performance with a small effect size after GABA supplementation. The increased activity of the sympathetic nervous system may be one of the reasons for improved functional firefighting balance performance.

## 1. Introduction

Firefighting is a highly hazardous and physically demanding occupation compared with many other professions. During firefighting operations, firefighters are routinely required to perform search-and-rescue tasks and transport injured individuals in highly unpredictable environments [1]. Under such conditions, maintaining postural stability and optimal balance represents a critical challenge. Accordingly, to better characterize firefighters’ functional firefighting balance abilities, previous studies have sought to develop balance assessment methods that are specific to firefighting-related tasks [2,3]. Therefore, evaluating functional firefighting balance while firefighters are wearing full protective gear under conditions that closely simulate real-world fireground scenarios is essential for occupational safety and operational efficiency.

However, certain operational or physiological conditions may compromise firefighting performance and consequently increase mission-related risks. For example, firefighters are required to wear personal protective equipment (PPE), self-contained breathing apparatuses (SCBAs), and protective face shields to minimize personal injury when operating in high-temperature, smoke-filled, low-visibility, and highly unpredictable environments. Previous studies have shown that such equipment may impair dynamic balance and increase the risk of injury during firefighting operations [2,3]. In addition to equipment-related constraints, physiological interventions intended to support recovery may also influence operational performance. GABA supplementation, through either dietary sources or nutritional supplements, has been reported to reduce work-related stress and improve sleep quality, particularly among individuals working shift schedules [4,5]. However, previous evidence has suggested that high-dose oral GABA supplementation may cross the blood–brain barrier and potentially impair cognitive function [6]. Furthermore, GABA supplementation has been reported to stimulate the vagus nerve through the enteric nervous system, which may enhance parasympathetic activity [7]. The sympathetic nervous system plays an important role in the execution of complex motor skills [8]. Conversely, increased parasympathetic activity may reduce physiological readiness and potentially compromise the performance of complex motor tasks. Therefore, acute GABA supplementation may enhance parasympathetic activity and, in turn, compromise functional firefighting balance.

One potential strategy to attenuate the adverse effects on cognitive function or functional firefighting balance associated with wearing personal protective equipment (PPE) or ingesting GABA may be to enhance sympathetic nervous system activity. Among the supplements known to influence cognitive function, motor skills, and sympathetic nervous system activity, caffeine is one of the most widely used ergogenic aids.

Recent studies have shown that chewing caffeinated gum containing caffeine at a dose of 3 mg/kg for 10 min results in peak salivary caffeine concentrations approximately 15 min after chewing [9,10]. Previous research has also used HRV in combination with salivary α-amylase concentration to examine the effects of caffeinated chewing gum on sympathetic nervous system activity. These findings suggest that caffeinated chewing gum can increase sympathetic nervous system activity and enhance exercise performance [10,11]. Compared to coffee or energy drinks, caffeine delivery through caffeinated chewing gum may accelerate absorption and reduce caffeine-related diuretic effects [12] and gastrointestinal discomfort [13].

However, whether caffeinated chewing gum can improve functional firefighting balance, particularly following the ingestion of a high dose of GABA, remains unclear. Therefore, the aim of the present study was to investigate whether caffeinated chewing gum enhances sympathetic nervous system activity and functional firefighting balance performance in elite firefighters wearing full protective gear after ingesting a high dose of GABA.

## 2. Materials and Methods

### 2.1. Study Design

**Figure 1:**
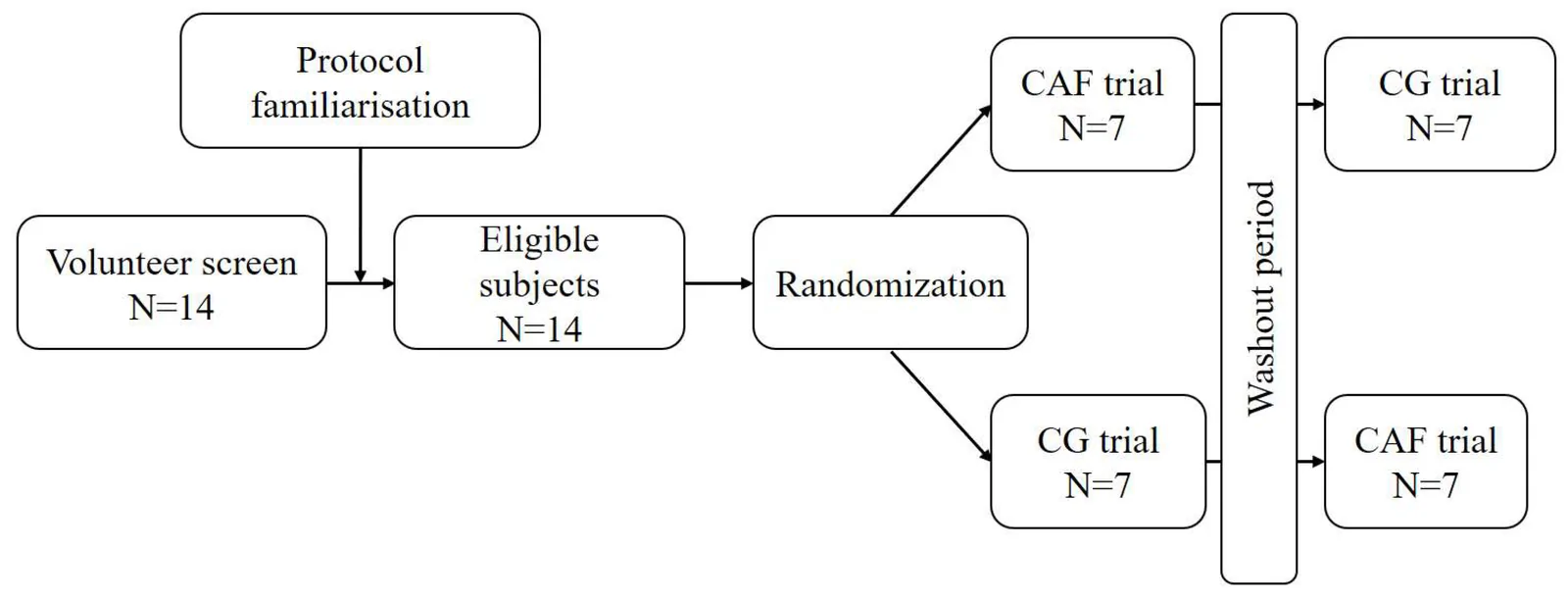
Study flow diagram of the randomized crossover trial. CAF, caffeine trial; CG, caffeine-free gum trial.

This study used a randomized crossover design with a double-blind experimental design, randomly assigning participants to either the caffeinated chewing gum trial (CAF) or the caffeine-free gum trial (CG). Participants completed the test in the evening following at least a one-day break to minimize the accumulated effects of consecutive shifts. After completing the first experiment, the participants underwent a 7- to 10-day washout period before the next experiment. This washout period was based on previous studies indicating that a 7- to 10-day washout period following high-intensity exercise is sufficient to achieve full physiological recovery for participants [9]. The experimental order was randomized using Excel software. First, the participants were assigned numbers, and their order was determined using a randomization sequence. Consequently, half of the participants (n = 7) were allocated to do the CAF trial; then the other half (n = 7) started with the CG trial (Figure 1). During the experiment, all participating firefighters continued their regular duties, with no changes to their work schedules or physical training. Each participant underwent two simulated functional firefighting balance tests prior to the formal experiment, performing 8–10 repetitions of each test to minimize motor learning effects. This study commenced on 1 July 2025 and concluded on 30 September 2025.

### 2.2. Participants

Fourteen active-duty elite male firefighters (age: 36 ± 5 years; height: 174 ± 5 cm; BMI: 25.2 ± 1.6 kg/m^2^) were recruited for and participated in this study. All participants were members of the Special Search and Rescue Team, Taiwan’s elite firefighting unit. The inclusion criteria for this study were as follows: (a) passed a special search and rescue program, (b) regularly carry out firefighting and rescue operations, and (c) recovery from sports injuries such as strains and sprains for more than three months. At the time of this study, the participants reported no symptoms such as stiffness or pain. The exclusion criteria were as follows: (a) suffering from epilepsy, hypertension, hyperlipidemia, heart disease, joint disorders, osteoporosis or brain injuries; (b) has no history of regular medication use; and (c) a history of caffeine allergy. All experimental procedures and associated risks were explained prior to the experiment. The experiment was conducted after each participant signed the informed consent form. All participants provided written informed consent before participation. This study received approval from the Institutional Review Board of Jen-Ai Hospital-Dali Branch (202400046B0D001) and retrospective registered in ClinicalTrials.gov (Date: 28 April 2026; ID “NCT07570030”; https://clinicaltrials.gov/study/NCT07570030?cond=NCT07570030&viewType=Card&rank=1 (accessed on 28 April 2026)). This study was conducted following the Declaration of Helsinki.

### 2.3. Sample Size Calculation

The present study used G*Power software (version 3.1.9.4, Universität Düsseldorf, Germany) [14] to calculate the requirements of the participants. There was no previous study examining the effects of supplementation of caffeine on balance performance in specific sports. This study used salivary α-amylase, a marker of sympathetic nervous system activation, as the basis for calculating the sample size. Previous study findings indicated that chewing the same dose of caffeinated gum significantly increased salivary α-amylase concentrations in special-forces soldiers with an effect size of 0.72 [10]. The software calculation was performed with the alpha level set at 0.05 and the correlation coefficient set at 0.80. The results showed that 14 participants were sufficient for the sample size.

### 2.4. Protocol

All formal experiments were conducted on the participants’ break day. After at least 8 h of sleep, the participants recorded the contents of their three meals prior to the formal experiment and consumed the same foods before the next experiment. Twenty-four hours before the experiment, the participants were required to completely abstain from consuming any beverages or foods containing caffeine, including coffee, energy drinks, tea, chocolate, or cola. Of the 14 participants, 2 were smokers. These 2 participants were required to refrain from smoking after 12:00. Three days before the official experiment, daily caffeine intake was recorded for each participant. Six participants (6/14, 42.9%) regularly consumed caffeine, with an average caffeine intake of 1.31 ± 0.35 mg/kg.

After finishing dinner at approximately 5:00 p.m., the participants rested in the laboratory for two hours. During this time, participants were free to read, watch TV, play video games, or engage in other non-physical activities, but were not allowed to sleep. At approximately 7:00 p.m., saliva samples were collected from the participants, and heart rate monitors were placed (Polar S80, Kempele, Finland) on all participants’ chests. The baseline resting heart rate and heart-rate variability then were measured by a 10 min analysis. Subsequently, the participants performed the functional firefighting balance test while wearing approximately 25 kg of full firefighting gear, including an oxygen cylinder. The participants completed a total of eight repetitions, with a one-minute rest in each set. After the test, the participants rested for one hour and were asked to take an 800 mg commercial GABA capsule (PharmaGABA^®^; 89% purity; Pharma Foods International Co., Ltd., Kyoto, Japan) and rest for 15 min. The participants then chewed either gum containing 3 mg/kg of caffeine (CAF trial) or caffeine-free gum (CG trial) for 10 min, spat it out, and rested for 15 min. The plasma concentration of GABA reaches its peak 30–45 min after ingestion [6]. Blood caffeine concentrations peak 15 min after chewing caffeinated gum [15]. This protocol was designed to achieve peak blood levels of GABA and caffeine [4,6,15]. After the chewing of the gum during the main trials, we asked the participants to guess which trial they had been assigned for the day. In the CAF trial, three participants (3/14; 21.4%) correctly guessed which trial they had participated in, four guessed incorrectly (4/14; 28.6%), and seven (7/14; 50%) answered “I don’t know.” In the CG trial, four participants (4/14; 28.6%) correctly guessed which trial they had participated in, four guessed incorrectly (4/14; 28.6%), and six (6/14; 42.9%) answered “I don’t know.”

The participants completed the functional firefighting balance test again, with heart rate and heart-rate variability analyzed throughout the test. After the test was completed, the participants were each asked to collect another saliva sample to complete the experimental trial.

### 2.5. Outcome Measure

#### 2.5.1. Functional Firefighting Balance Test

The functional firefighting balance tests have been shown in a previous study [16] to indicate sufficient validity and reliability for measuring firefighters’ balance ability and serving-as-firefighting performance. Participants underwent the test while wearing full firefighting gear and carrying a breathing apparatus and face mask. In the test area, a wooden platform measuring 1 m in length and width and 20 cm in height was set up. Participants were asked to walk along a narrow wooden beam (4 m long, 15 cm wide, and 5 cm high), to pass under an overhead obstacle to reach the wooden platform on the opposite side, and then to walk backward as quickly as possible to return to the starting wooden platform. A staff member placed a light wooden stick on a narrow wooden beam at a height equal to 75% of each firefighter’s height. When the firefighter touched the stick, it fell to the ground, and the staff member picked it up and put it back in place. Time gates (Microgate^®^, Whitty, Italy) were used to record the participants’ completion times, and two staff members recorded the number of errors made by the participants. Major errors included (a) dropping the wooden stick; (b) both feet touching the ground; and (c) the subject falling during the test. Minor errors included: (a) a hand or foot touching the ground; (b) a hand touching the wooden platform; and (c) the subject touching the wooden stick but the stick not falling. We added 1 s to the completion time for minor errors and 2 s for major errors and included these times in the total completion time. This experiment recorded the average completion time and the performance index (PI) for eight repetitions. The formula for calculating the performance index is PI = completion time (seconds) + (times of minor errors × 1) + (times of major errors × 2).

#### 2.5.2. Heart-Rate Variability (HRV) and Saliva Sample Collection and Analysis

The HRV measurement and analysis methods used in the current study have been shown in previous studies [17,18]. A portable heart rate monitor (Polar S80, Kempele, Finland) was used to record the beat-to-beat heart rate at a frequency of precisely 1 ms. In addition to securing the device to the chest with the original strap, this study used athletic tape to further stabilize it. At baseline, the total recording time was 10 min. During the functional firefighting balance test, the time recorded was from the start of the first set to the end of the last set: approximately 10 min. The analysis utilized time-domain measures including the percentage of successive RR interval differences, the standard deviation of NN intervals (SDNN), and the root mean square of successive differences (RMSSD), as well as frequency-domain components, specifically normalized low-frequency (LF) and high-frequency (HF) units. The LF/HF ratio was calculated as the ratio of normalized low frequency to high frequency. HRV data were processed using Kubios HRV Scientific software (v3.0; Kubios Oy, Kuopio, Finland). Artifacts were addressed using the medium-level threshold filter (set at 0.25 s) to ensure data integrity [19]. The same methods for securing HRV devices and analyzing the data, particularly in exercise conditions, have already been reported in a previous study [17].

Saliva samples were collected using sterile plastic saliva collection tubes. After collecting approximately 2 mL of saliva, it was immediately stored in a −80 °C freezer until further analysis. The saliva samples were thawed and centrifuged at 4000 rpm for 5 min. Subsequently, 500 μL aliquots were transferred to glass tubes for an enzyme-linked immunosorbent assay (ELISA) using commercial kits (Neogen Corporation, Lexington, KY, USA; Salimetrics LLC, State College, PA, USA) for saliva α-amylase concentrations. All samples were assayed in triplicate, with CVs for α-amylase of 5.2%.

### 2.6. Caffeine and Caffeine-Free Gum

In this study, commercially available caffeinated chewing gum (Military Energy Gum, Arctic Mint flavor; Stay Alert, Chicago, IL, USA) was weighed and crushed, then mixed with mint-flavored chewing gum (Lotte Gum Mint., Ltd., Tokyo, Japan) and 0.3 g of peppermint flavoring powder. According to the product packaging, each 5 g piece of gum contains 100 mg of caffeine. The mixture was reshaped to produce caffeinated gum containing 3 mg/kg. The caffeine-free gum was prepared using the same mint-flavored chewing gum, which was crushed, mixed with 0.3 g of peppermint flavoring powder, and reshaped to match the caffeinated gum in appearance, texture, and flavor. All gum was produced by staff members who were not involved in participant recruitment, data collection, or outcome assessment and were labeled using a coded system so that the study investigators and participants were unaware of the treatment allocation on each trial day (double-blind design) [9,20,21].

### 2.7. Statistical Analysis

All data are presented as means ± standard deviations. All data were tested for normality using the Shapiro–Wilk test. The paired *t*-test was used to analyze the baseline parameters between the two trials and the results of the functional firefighting balance test, including completion time and performance index, after GABA supplementation. The HRV (baseline and during test) and salivary α-amylase (baseline and after tests) were analyzed using two-way repeated-measures ANOVA, followed by Bonferroni post hoc tests to determine significant differences between conditions and time points. Effect sizes were calculated using Cohen’s d and interpreted according to the following thresholds: negligible (<0.20), small (0.20–0.59), medium (0.60–1.19), large (1.20–1.99), and very large (≥2.00) [22]. Significance was set at α < 0.05.

## 3. Results

### 3.1. Baseline Parameters

There were no significant differences between the CAF and CG trials in HR (bpm), SDNN (ms), RMSSD (ms), LF (n.u.), HF (n.u.), LF/HF, completion time, performance index and saliva α-amylase concentration (Table 1).

### 3.2. Functional Firefighting Balance Test

**Figure 2:**
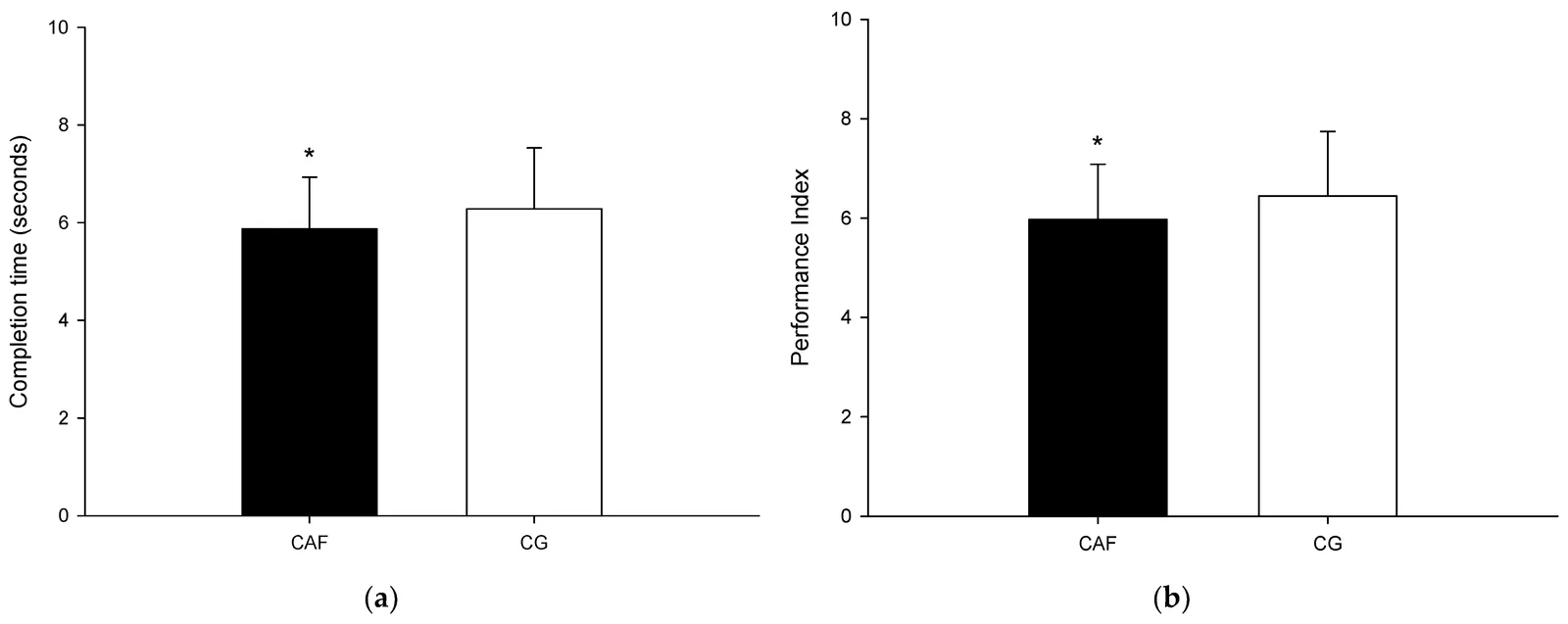
Effects of caffeinated chewing gum on functional firefighting balance performance: (**a**) completion time and (**b**) performance index. Values are presented as means ± SD. CAF, caffeine trial; CG, caffeine-free gum trial. * *p* < 0.05 compared with CG.

After GABA supplementation, there were significantly better completion times (mean difference [95% confidence interval]: 0.41 [0.11, 0.72]; *p* = 0.011; Cohen’s d = 0.54; Figure 2a) and PIs (mean difference [95% confidence interval]: 0.48 [0.12, 0.83]; *p* = 0.012; Cohen’s d = 0.43; Figure 2b), with smaller effect sizes in the CAF trial than those of the CG trial.

### 3.3. Heart-Rate Variability, Saliva α-Amylase Concentration

Table 2 summarizes the results for the heart-rate variability and salivary α-amylase concentrations. There were no significant interactions in HR, SDNN, RMSSD, LF or HF (condition*time, *p* > 0.05). There was a significant interaction in LF/HF (*p* = 0.040); however, further statistical analysis revealed no significant differences across trials or over time (*p* > 0.05). There was a significant interaction in saliva α-amylase concentration (condition × time, *p* = 0.018). Post hoc analysis revealed that there was a significantly higher saliva α-amylase concentration in the CAF trial (mean difference [95% confidence interval]: 38.7 [0.7, 96.6]; *p* = 0.048; Cohen’s d = 0.45) compared with that of the CG trial, with small effect sizes after tests.

## 4. Discussion

The present study indicates that the ingestion of caffeinated chewing gum containing 3 mg/kg of caffeine after oral supplementation of GABA significantly improved completion time and the performance index (PI) in the functional firefighting balance test. The elevated salivary α-amylase concentrations after the functional firefighting balance tests indicate that caffeinated chewing gum may effectively activate the sympathetic nervous system (SNS). The increased activity of the sympathetic nervous system may be one of the reasons for the improved functional firefighting balance performance.

Using firefighting equipment [2,3] or the supplementation of GABA [8] by firefighters may compromise functional firefighting balance. Although this study did not include a control trial to determine whether firefighting equipment or GABA supplementation affected firefighters’ functional balance, the present study has indicated that the ingestion of caffeinated chewing gum effectively improved functional firefighting balance in elite firefighters. Although numerous studies have shown that caffeine supplementation enhances various exercise performances [23], there are only a small number of studies that have examined topics related to postural balance [24]. None of these studies have specifically investigated balance in relation to functional sport-specific performance. Therefore, the present study is the first study to examine the relationship between caffeine supplementation and balance ability in a specific exercise. The findings from the present study indicate the aforementioned results when with the use of firefighting equipment and under conditions involving prior GABA supplementation, caffeinated chewing gum can improve functional balance in firefighters.

The increase in sympathetic nervous system activity induced by caffeinated chewing gum may be one of the possible mechanisms for the improved functional firefighting balance ability observed in the elite firefighters in this study. It has been found that activation of the sympathetic nervous system enhances motor control during complex motor control [10]. A previous study has found that the enhancement of sympathetic nervous system activity induced by caffeine significantly improved cognitive function and physical performance [10]. In this study, although there was no significant difference in heart-rate variability, the increased salivary α-amylase concentrations indicate that caffeinated chewing gum effectively activates the sympathetic nervous system. These data suggest that higher sympathetic nervous system activation may be one of the possible reasons for the higher functional firefighting balance test scores in the CAF trial.

Caffeinated chewing gum significantly increased sympathetic nervous system activity with a small effect size. Similarly, its impact on functional firefighting balance tests scores were also small. A previous study has found that the absolute strength of isometric leg extension and flexion may be a factor affecting the functional firefighting balance ability of firefighters [16]. It has been clearly shown that a moderate to small effect size of caffeine on absolute muscle strength has been observed in a previous review study [24]. In addition, a previous study has found similarly that caffeinated chewing gum showed medium-to-large effect sizes on indicators of sympathetic nervous system activation but only small effect sizes on cognitive function, strength and power performance [10]. During functional firefighting balance tests performed while carrying approximately 25 kg of equipment, lower-body muscle strength may have played an important role in functional firefighting balance ability. The effect size of caffeinated chewing gum on absolute muscle strength is small, which may explain why the impact on functional firefighting balance tests was also relatively small.

Although the statistical results indicate a small effect size on functional firefighting balance performance, in practical application, particularly during high-risk rescue operations by elite firefighters, even minor changes may reduce injury risk. The firefighting balance test used in this study was designed to simulate situations encountered during search and rescue missions, such as walking on uneven ground and avoiding overhead obstacles. Maintaining proper body posture in such situations may reduce the risk of slips, trips, and falls [17]. In addition, when firefighters receive a duty notification, the preparation time may be as short as 5 min, and they must arrive on site as quickly as possible. Caffeine intake via energy drinks or coffee may not be suitable during fire engine operations. Not only may the caffeine dose be insufficient, but it also takes approximately one hour after ingestion for blood caffeine levels to peak [24], which may be too slow to produce an adequate effect during rescue operations. In this situation, caffeinated gum can serve as a good alternative. It is absorbed more quickly [9,10] and is convenient to chew while on a fire truck.

Prior to this experiment, G*Power software was used to conduct a sample size calculation, and it was determined that the sample size for this study was sufficient to ensure reliable data interpretation. With regard to indicators of functional firefighting balance performance, caffeinated chewing gum was associated with a small effect size. Therefore, there is a possibility that the observed effects of caffeinated chewing gum on functional firefighting balance performance represent a Type II error [25]. However, regarding sympathetic nervous system activity, G*Power analysis revealed that for statistical power for salivary α-amylase, the calculated power value was 0.8, indicating a low probability of a Type II error [26]. These results suggest that caffeinated chewing gum has an effect on sympathetic nervous system activity, but the findings for firefighting balance performance may reflect a Type II error and warrant further study.

### Strengths and Limitations

The rigorous methodological framework employed in this study enhances the robustness of its findings. In addition, this study represents the first systematic investigation of the effects of caffeinated chewing gum-mediated sympathetic activation on functional firefighting balance performance in elite firefighters, highlighting its significant scientific novelty and contribution to the literature. There are several limitations in the current study. First, this study did not measure lower-body muscle strength or cognitive function, which makes it impossible to clearly identify the exact mechanisms by which sympathetic nervous system activation improves functional firefighting balance tests. Second, this study only recruited male firefighters as participants. The effects of caffeine on enhancing physical performance may vary by gender [27,28]. Therefore, the results of this study may not be directly applicable to female firefighters. In addition, this study was conducted by firefighters who had gotten sufficient sleep and performed the experiments in a controlled environment. The results may not necessarily be applicable to environments with high temperatures, thick smoke, or other similar conditions. The fire balance test used in this study may be subject to a risk of Type II error. In addition, the lack of a control group without GABA, possible learning effects, lack of respiratory control during HRV assessment, and retrospective trial registration are limitations that future studies could address. Finally, during this experiment, we did not assess potential order effects; therefore, it remains unclear whether the order of the experimental conditions influenced the participants’ performance. To evaluate the potential order effect, we analyzed the firefighting balance test results following GABA supplementation according to the participants’ experimental order. No significant differences were observed between the two groups in completion time (*p* = 0.72) or performance index (*p* = 0.84), indicating that the order of the experimental conditions did not significantly influence the study outcomes.

## 5. Conclusions

This study suggests that caffeine administered via caffeinated chewing gum may improve functional firefighting balance performance in elite firefighters wearing full firefighting gear after GABA supplementation. The study data also showed increases in markers of sympathetic nervous system activity, which may be one of the reasons for the improvement in functional balance performance.

## Figures and Tables

**Table 1:** Baseline parameters.

	CAF	CG	*p*
HR (bpm)	80 ± 14	82 ± 14	0.513
SDNN (ms)	28.3 ± 11.2	31.3 ± 13.3	0.375
RMSSD (ms)	22.9 ± 10.7	28.5 ± 15.1	0.251
LF (n.u.)	72.5 ± 8.8	67.4 ± 13.8	0.280
HF (n.u.)	27.5 ± 8.8	32.5 ± 13.8	0.281
LF/HF	2.6 ± 0.8	2.6 ± 1.6	0.886
Completion time (seconds)	6.3 ± 1.2	6.5 ± 1.2	0.285
Performance Index	6.5 ± 1.2	6.6 ± 1.3	0.335
Saliva α-amylase (U/mL)	55.7 ± 41.8	59.3 ± 34.6	0.716

Values are means ± SD, n = 14. CAF, caffeine trial; CG, caffeine-free gum trial.

**Table 2:** The heart-rate variability and α-amylase concentrations.

	CAF		CG		Condition × Time		Condition		Time	
	*Baseline*	*During test*	*Baseline*	*During test*	*p value*	*η*2	*p value*	*η*2	*p value*	*η*2
HR (bpm)	80 ± 14	99 ± 18	82 ± 14	100 ± 11	0.754	0.008	0.625	0.019	<0.001	0.747
SDNN (ms)	28.3 ± 11.2	34.2 ± 13.9	31.3 ± 13.3	33.6 ± 13.2	0.830	0.006	0.918	0.001	0.519	0.054
RMSSD (ms)	22.9 ± 10.7	28.7 ± 15.8	28.5 ± 15.1	30.3 ± 13.6	0.831	0.005	0.479	0.057	0.615	0.029
LF (n.u.)	72.5 ± 8.8	77.0 ± 9.5	67.4 ± 13.8	68.4 ± 10.2	0.356	0.066	0.045	0.275	0.251	0.100
HF (n.u.)	27.5 ± 8.8	22.9 ± 9.5	32.5 ± 13.8	31.5 ± 10.2	0.355	0.066	0.042	0.274	0.246	0.102
LF/HF	2.6 ± 0.8	3.7 ± 1.7	2.5 ± 1.5	2.6 ± 1.5	0.040	0.234	0.121	0.188	0.260	0.104
	*Baseline*	*After test*	*Baseline*	*After test*						
Saliva α-amylase (U/mL)	55.7 ± 41.8	129.7 ± 96.4 *	59.3 ± 34.6	91.1 ± 67.3	0.018	0.411	0.048	0.146	0.004	0.552

Values are means ± SD, n = 14. CAF, caffeine trial; CG, caffeine-free gum trial. * Value for CAF was significantly different than that for CG.

## Data Availability

The data of the current study are available on reasonable request from the corresponding author.

## References

[1] Williams-Bell F.M., CMcGregor A.M. (2022). Firefighter Personnel and Their Activities in Extreme Environments, in Engineering and Medicine in Extreme Environments.

[2] Rosengren K.S., Hsiao-Wecksler E.T., Horn G. (2014). Fighting fires without falling: Effects of equipment design and fatigue on firefighter’s balance and gait. Ecol. Psychol..

[3] Brown M.N., Char R.M.M.L., Henry S.O., Tanigawa J., Yasui S. (2019). The effect of firefighter personal protective equipment on static and dynamic balance. Ergonomics.

[4] Yamatsu A., Yamashita Y., Pandharipande T., Maru I., Kim M. (2016). Effect of oral γ-aminobutyric acid (GABA) administration on sleep and its absorption in humans. Food Sci. Biotechnol..

[5] Hou D., Tang J., Feng Q., Niu Z., Shen Q., Wang L., Zhou S. (2024). Gamma-aminobutyric acid (GABA): A comprehensive review of dietary sources, enrichment technologies, processing effects, health benefits, and its applications. Crit. Rev. Food Sci. Nutr..

[6] Lim L.W., Aquili L. (2021). GABA supplementation negatively affects cognitive flexibility independent of tyrosine. J. Clin. Med..

[7] Almutairi S., Sivadas A., Kwakowsky A. (2024). The effect of oral GABA on the nervous system: Potential for therapeutic intervention. Nutraceuticals.

[8] Collet C., Roure R., Delhomme G., Dittmar A., Rada H., Vernet-Maury E. (1999). Autonomic nervous system responses as performance indicators among volleyball players. Eur. J. Appl. Physiol..

[9] Shiu Y.-J., Chen C.-H., Tao W.-S., Nai H.-F., Yu C.-Y., Chiu C.-H. (2024). Acute ingestion of caffeinated chewing gum reduces fatigue index and improves 400-meter performance in trained sprinters: A double-blind crossover trial. J. Int. Soc. Sports Nutr..

[10] Shiu Y.-J., Chen C.-H., Lin H.-Y., Yang C.-C., Yen C.-W., Chang H.-Y., Chen H.-T., Chiu C.-H. (2026). Caffeinated chewing gum improves military performances in special forces soldiers. Eur. J. Appl. Physiol..

[11] Tzeng G.-J., Lin H.-Y., Shiu Y.-J., Hsieh M.-H., Chen Z.-C., Chiu C.-H. (2026). Caffeinated Chewing Gum Improves Sympathetic Nerve Activity and Wrestling Performance: A Double-Blind Crossover Trial. Int. J. Sports Physiol. Perform..

[12] Zhang Y., Coca A., Casa D.J., Antonio J., Green J.M., Bishop P.A. (2015). Caffeine and diuresis during rest and exercise: A meta-analysis. J. Sci. Med. Sport.

[13] Ruiz-Moreno C., Lara B., Salinero J.J., de Souza D.B., Ordovás J.M., Del Coso J. (2020). Time course of tolerance to adverse effects associated with the ingestion of a moderate dose of caffeine. Eur. J. Nutr..

[14] Faul F., Erdfelder E., Lang A.-G., Buchner A. (2007). G* Power 3: A flexible statistical power analysis program for the social, behavioral, and biomedical sciences. Behav. Res. Methods.

[15] Kamimori G.H., Karyekar C.S., Otterstetter R., Cox D.S., Balkin T.J., Belenky G.L., Eddington N.D. (2002). The rate of absorption and relative bioavailability of caffeine administered in chewing gum versus capsules to normal healthy volunteers. Int. J. Pharm..

[16] Mota J.A., Barnette T.J., Gerstner G.R., Giuliani H.K., Tweedell A.J., Kleinberg C.R., Thompson B.J., Pietrosimone B., Ryan E.D. (2018). Relationships Between Neuromuscular Function and Functional Balance Performance in Firefighters. Sci. Rep..

[17] Liu H.-S., Okada T., Mitsuya H., Hsieh M.-H., Hou C.-W., Chang C.-C., Chiu C.-H. (2025). Effects of caffeinated chewing gum-induced sympathetic activation and diuretic effect on the rapid rate of weight loss in bodybuilders: A double-blind crossover study. Bmc Sports Sci. Med. Rehabil..

[18] Hung B.-L., Chen L.-J., Chen Y.-Y., Ou J.-B., Fang S.-H. (2021). Nicotine supplementation enhances simulated game performance of archery athletes. J. Int. Soc. Sports Nutr..

[19] Lipponen J.A., Tarvainen M.P. (2019). A robust algorithm for heart rate variability time series artefact correction using novel beat classification. J. Med. Eng. Technol..

[20] Wu S.-H., Chen Y.-C., Chen C.-H., Liu H.-S., Liu Z.-X., Chiu C.-H. (2024). Caffeine supplementation improves the cognitive abilities and shooting performance of elite e-sports players: A crossover trial. Sci. Rep..

[21] Liu H.-S., Liu C.-C., Shiu Y.-J., Lan P.-T., Wang A.-Y., Chiu C.-H. (2024). Caffeinated chewing gum improves basketball shooting accuracy and physical performance indicators of trained basketball players: A double-blind crossover trial. Nutrients.

[22] Hopkins W.G. A Scale of Magnitudes for Effect Statistics, 2002. http://www.sportsci.org/resource/stats/index.html.

[23] Guest N.S., VanDusseldorp T.A., Nelson M.T., Grgic J., Schoenfeld B.J., Jenkins N.D.M., Arent S.M., Antonio J., Stout J.R., Trexler E.T. (2021). International society of sports nutrition position stand: Caffeine and exercise performance. J. Int. Soc. Sports Nutr..

[24] Briggs I., Chidley J.B., Chidley C., Osler C.J. (2021). Effects of caffeine ingestion on human standing balance: A systematic review of placebo-controlled trials. Nutrients.

[25] Banerjee A., Chitnis U., Jadhav S., Bhawalkar J., Chaudhury S. (2009). Hypothesis testing, type I and type II errors. Ind. Psychiatry J..

[26] Akobeng A.K. (2016). Understanding type I and type II errors, statistical power and sample size. Acta Paediatr..

[27] Temple J.L., Ziegler A.M. (2011). Gender differences in subjective and physiological responses to caffeine and the role of steroid hormones. J. Caffeine Res..

[28] Ouergui I., Delleli S., Bridge C.A., Messaoudi H., Chtourou H., Ballmann C.G., Ardigò L.P., Franchini E. (2023). Acute effects of caffeine supplementation on taekwondo performance: The influence of competition level and sex. Sci. Rep..

